# Targeting gut microbiota for diabetic nephropathy treatment: probiotics, dietary interventions, and fecal microbiota transplantation

**DOI:** 10.3389/fendo.2025.1621968

**Published:** 2025-06-30

**Authors:** Xiaoran Wang, Xinyin Liu, Fanghong Gong, Yan Jiang, Canwei Zhang, Wei Zhou, Wen Zhang

**Affiliations:** ^1^ Department of Nephrology, The First People’s Hospital of Hangzhou Lin’an District, Hangzhou, China; ^2^ Department of Traditional Chinese Medicine, Jiande First People’s Hospital, Jiande, Hangzhou, China; ^3^ Department of Nephrology, The First Affiliated Hospital of Zhejiang Chinese Medical University (Zhejiang Provincial Hospital of Chinese Medicine), Zhejiang Key Laboratory of Research and Translation for Kidney Deficiency-Stasis-Turbidity Disease, Zhejiang-Macau International Joint Laboratory of Integrated Traditional Chinese and Western Medicine for Nephrology and Immunology, Hangzhou, China

**Keywords:** diabetic kidney disease, gut microbiota, short-chain fatty acids (SCFAs), probiotics, fecal microbiota transplantation (FMT)

## Abstract

Diabetic nephropathy (DN) stands as a prominent microvascular complication of diabetes mellitus and presents a significant global health challenge. Despite advancements in glycemic control and renin-angiotensin system inhibition, current treatments merely delay disease progression without targeting fundamental pathological processes. This review explores gut microbiota modulation as a promising treatment strategy for DN through probiotic supplementation, dietary interventions, and fecal microbiota transplantation(FMT) protocols. The gut microbiota, integral to the “gut-kidney axis,” is critically implicated in DN pathogenesis. DN is associated with gut dysbiosis—characterized by reduced microbial diversity, depletion of beneficial short-chain fatty acid (SCFA)-producing bacteria, and proliferation of opportunistic pathogens. This dysbiosis impairs gut barrier integrity, fostering systemic inflammation and the accumulation of uremic toxins like indoxyl sulfate. Furthermore, translocated bacterial lipopolysaccharides activate Toll-like receptors and the NLRP3 inflammasome, exacerbating kidney damage and fibrosis. Interventions targeting the microbiota, including dietary strategies (e.g., enhancing fermentable fibers, low-protein diets) and FMT, show promise in preclinical and early clinical studies, though FMT requires stringent safety and donor screening protocols. Significant challenges persist, such as managing inter-individual microbiota variability for personalized therapies, fully elucidating molecular mechanisms like SCFA-GPR43 signaling, and leveraging multiomics for biomarker discovery. Advancing microbiota-focused interventions for DN towards microbiome-centered precision medicine necessitates addressing standardization, deepening mechanistic understanding, and validating combination therapies, heralding a potential shift from traditional nephroprotective approaches.

## 1 Introduction

Diabetic nephropathy (DN) is a severe microvascular complication of diabetes mellitus and is the leading cause of end-stage renal disease (ESRD) worldwide, accounting for approximately 40% of ESRD cases (1, 2). The increasing global prevalence of diabetes has led to a corresponding increase in the incidence of DN, which presents a pressing clinical challenge.

Histologically, DN is characterized by progressive glomerulosclerosis, tubulointerstitial fibrosis, and abnormal extracellular matrix deposition, resulting in irreversible nephron loss and functional decline (3, 4). Although intensive glycemic control and renin-angiotensin system RAS inhibition remain therapeutic cornerstones, they predominantly decelerate clinical deterioration rather than halting disease progression. Notably, these interventions show limited capacity to repair existing renal structural damage, especially in late-stage nephropathy (3, 5). This significant gap underscores the urgent need to both investigation of new pathogenic mechanisms and development of targeted therapies. The gut-kidney axis now constitutes a critical framework for understanding DN pathophysiology. This bidirectional communication network is characterized by complex interactions involving microbial metabolites, immune system regulation, and inflammatory pathways (6, 7). Supporting this concept, clinical investigations consistently demonstrate characteristic microbial imbalances in DN patients. Key features include reduced microbial ecological diversity, decreased beneficial butyrate-producing bacteria (e.g., Roseburia and Faecalibacterium), increased lipopolysaccharide (LPS)-producing gram-negative organisms (notably Enterobacteriaceae), and elevated urease-producing microbes (7–10). These alterations compromise intestinal barrier integrity, facilitating bacterial endotoxin translocation and toll-like receptor 4-mediated pro-inflammatory cascades, thereby amplifying oxidative stress and fibrogenic responses within the renal parenchyma (3, 11, 12). Additionally, aberrant gut microbial metabolism contributes to peripheral insulin resistance and accelerates advanced glycation end product formation, exacerbating glomerular basement membrane thickening and podocyte dysfunction (8, 13, 14).

Microbiota-targeted therapeutic strategies hold considerable promise for the management of DN. Preclinical investigations have demonstrated that selective probiotic supplementation, particularly with *Lactobacillus* and *Bifidobacterium* species, or prebiotic administration, effectively restores microbial homeostasis, enhances colonic short-chain fatty acid (SCFA) production, and subsequently inhibits the TGF-β1/Smad3 signaling pathway in the kidneys (9, 11, 15). These interventions attenuate epithelial–mesenchymal transition in tubular epithelial cells and reduce pathological collagen deposition, which are key pathological features of DN (11, 15).

Translational studies and early phase clinical trials have indicated that targeted microbiota transplantation protocols and structured dietary fiber supplementation significantly reduce albuminuria, improve insulin sensitivity, and ameliorate dyslipidemia in patients (3, 16). Notably, specific gut microbiota-derived metabolites, such as indolepropionic acid and trimethylamine-N-oxide, correlate strongly with the severity of renal fibrosis, suggesting their potential as clinically relevant biomarkers for the early detection and therapeutic monitoring of DN management (3, 8, 17). Furthermore, targeted microbiome-based interventions may provide a promising translational approach to overcome current limitations in the management of DN. These novel therapeutic strategies have the potential to offer effective treatment options for clinically challenging complication (3, 18). This review examines therapeutic targeting of the gut microbiota in DN management through probiotics, dietary modifications, and FMT.

## 2 Molecular mechanisms by which the gut microbiota influences DN

### 2.1 Pathological interactions mediated by the gut–kidney axis via microbial metabolite imbalance

#### 2.1.1 SCFA depletion

Metabolic dysregulation and barrier disruption SCFAs, notably butyrate and propionate, modulate immune responses by activating G protein-coupled receptors (GPR43/41) and inhibiting histone deacetylase (HDAC) activity, leading to pro-inflammatory cytokine transcription suppression (19–21). These metabolites are crucial to maintain intestinal barrier integrity by enhancing the expression of tight junction proteins such as zona occludens-1, occludin, and claudin-1, and stimulating mucin production from goblet cells (4, 13, 22). In patients with DN, quantitative assessments reveal a 30–50% reduction in intestinal SCFA levels, which negatively correlates with gut permeability markers (22–24). Metagenomic analyses indicate significant reductions (2.5–3.8-fold) in key butyrate-producing bacteria, including *Faecalibacterium prausnitzii* and *Roseburia* species, in patients with DN patients compared to healthy controls (23–25). The functional relevance of these changes has been highlighted by interventional studies in which sodium butyrate supplementation (200 mg/kg/day) in diabetic C57BL mice with 14-day quercetin pretreatment restored colonic tight junction expression and decreased circulating lipopolysaccharide levels by 42% (26).

#### 2.1.2 Uremic toxin accumulation and nephrotoxicity mechanisms

**Figure 1:**
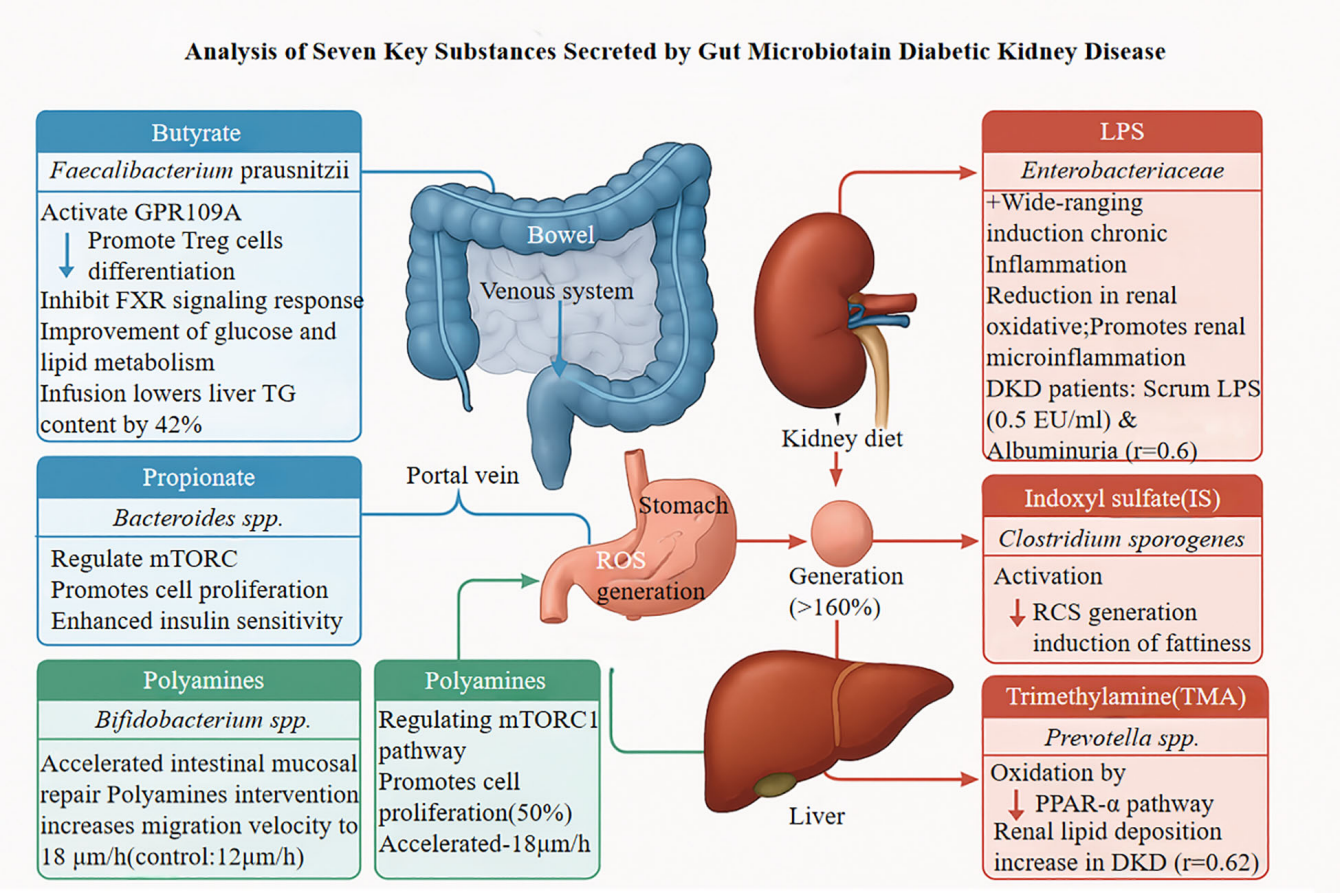
GRP109A, G Protein-Coupled Receptor 109A; FXR, Farnesoid X Receptor; TG, Triglycerides; LPS, Lipopolysaccharide; DKD, Diabetic Kidney Disease; TORC, Target of Rapamycin Complex; RCS, Reactive Carbonyl Species; TMA, Trimethylamine; PPAR-α, Peroxisome Proliferator-Activated Receptor α.

Protein-bound uremic toxins significantly contribute to the progression of DN. Indoxyl sulfate (IS), a tryptophan-derived bacterial metabolite, induces pyroptotic cell death in renal tubular cells through aryl hydrocarbon receptor activation, triggering oxidative stress, NLRP3 inflammasome assembly, and caspase-1 activation (21, 26–28). P-Cresyl sulfate (pCS) induces renal tubular cell injury through oxidative stress and direct cytotoxic effects. The anti-proliferative impact of pCS on renal cells surpasses that of other uremic toxins (e.g., indoxyl sulfate, IS), potentially accelerating kidney disease progression via pro-fibrotic signaling pathway activation (29). pCS upregulates renal fibrosis markers and disrupts repair mechanisms of tubular epithelial cells, characterized by reduced autophagic flux and mitochondrial dysfunction. Systemic accumulation of pCS, correlated with elevated gut microbiota-derived precursor p-cresol levels, further exacerbates renal function decline in chronic kidney disease (CKD) patients. The nephrotoxicity of pCS is potentially compounded by inhibition of r renal membrane transporters, such as organic anion transporters 1/3 (OAT1/OAT3). Such inhibition curtails the efficient excretion of uremic toxins (including pCS), thus perpetuating a vicious cycle of toxin accumulation, heightened systemic inflammation, and progressive renal injury (30).Additionally, IS and p-cresyl sulfate activate the pro-fibrotic TGF-β1/Smad3 pathway, increasing renal cortical TGF-β1 expression approximately 3.2-fold and promoting interstitial fibrosis, effects that are abrogated by selective AhR antagonism (18, 31, 32). The clinical relevance of these mechanisms is supported by significantly higher circulating IS concentrations in patients with stage 3–4 DN compared to non-nephropathic individuals with diabetes, and these elevated concentrations strongly correlate with the rate of eGFR decline (18, 28, 32). A randomized controlled trial (NCT04111775) demonstrated that oral charcoal adsorbent AST-120 reduces serum IS concentrations by 58% and decreases the urinary protein-to-creatinine ratio by 29% after 12 weeks, providing evidence that targeting gut-derived uremic toxins offers potential therapeutic benefits for established DN (28, 29). Analysis of the seven key metabolites secreted by the gut microbiota in patients with DN is presented in **Figure 1**.

### 2.2 Molecular mechanisms of toll-like receptor activation and therapeutic strategies

#### 2.2.1 TLR4 signaling in DN

Lipopolysaccharide (LPS), a key component of the outer membrane of gram negative bacteria, acts as a potent pathogen-associated molecule that triggers inflammatory responses. LPS recognition occurs through binding to membrane-bound or soluble CD14, forming a complex that interacts with the TLR4/MD-2 heterodimer in renal and immune cells (33). This interaction initiates a MyD88-dependent signaling cascade. MyD88, recruited to the activated receptor complex, functions as a scaffold for IL-1 receptor-associated kinases (IRAKs) and tumor necrosis factor receptor-associated factor 6 (TRAF6). The resulting proximal signaling complex activates inhibition of κB kinase (IKK), leading to phosphorylation and proteasomal degradation of inhibitory IκB proteins. This releases NF-κB p65 subunits, enabling their nuclear translocation and DNA binding (34, 35).

NF-κB activation upregulates pro-inflammatory mediators, including cytokines (IL-1β, IL-6, TNF-α) and chemokines (MCP-1), fostering a microenvironment that exacerbates kidney injury. The inflammatory response is self-amplifying, as NF-κB activation further increases TLR4 expression, creating a positive feedback loop (36). Efficient TLR4 signaling also depends on membrane microarchitecture modifications, such as lipid raft reorganization and the coordinated recruitment of adaptor proteins TIRAP/MAL and MyD88 to form multimeric signalosome (34, 37, 38).

#### 2.2.2 Experimental evidence supporting TLR4 involvement

**Figure 2:**
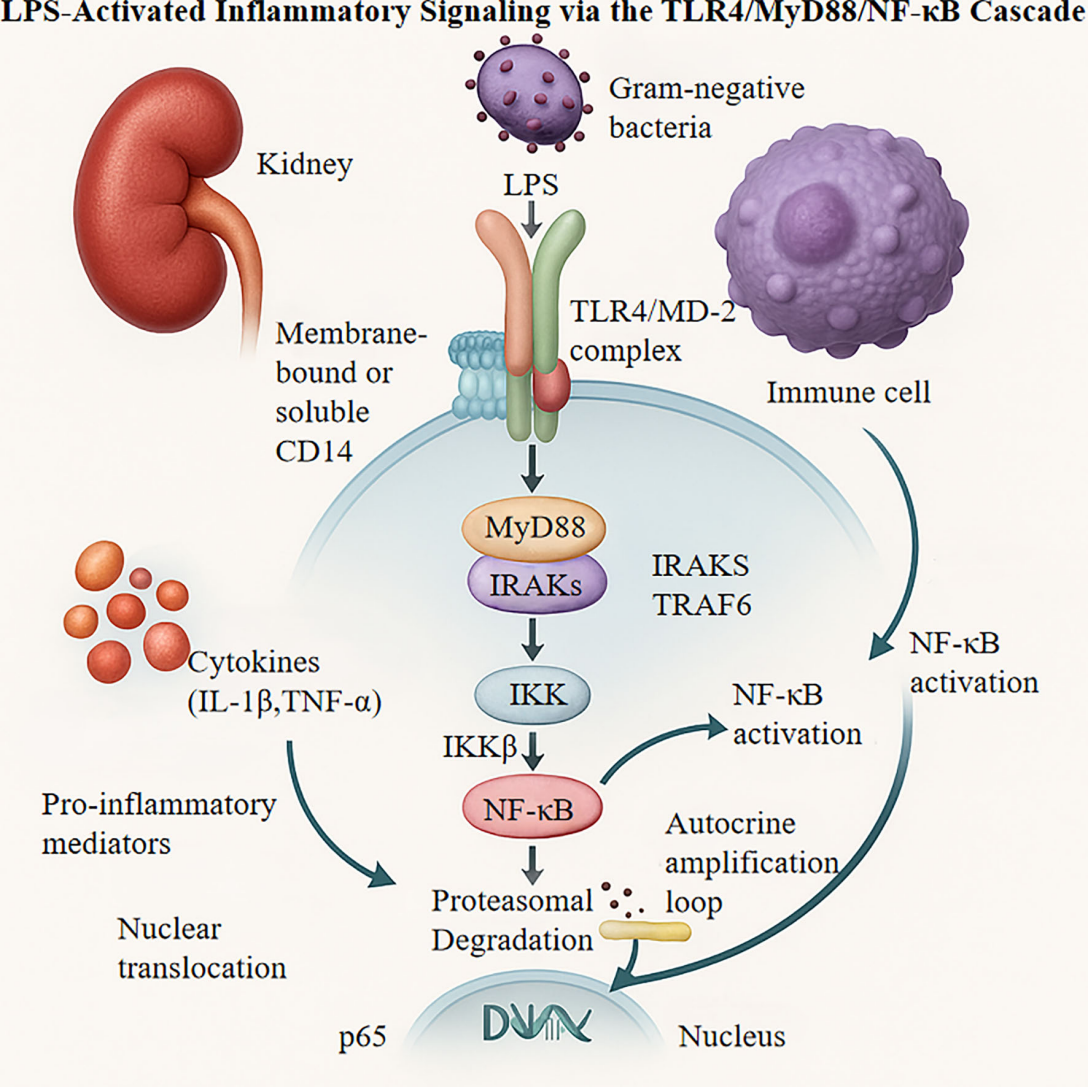
LPS, Lipopolysaccharide; TLR4, Toll-like Receptor 4; MD-2, Myeloid Differentiation Protein 2; IRAKS, IL-1 receptor-associated kinases; TRAF6, TNF Receptor Associated Factor 6; IKK, Inhibitor of NF-κB Kinase; MyD88, Myeloid Differentiation Primary Response Protein 88; CD14, Cluster of Differentiation 14.

*In vitro* studies using LPS-stimulated macrophages have demonstrated substantial temporal increases in TLR4 protein expression (3.2-fold within 6 hours; p < 0.01), accompanied by enhanced expression of downstream intermediates, particularly MyD88 (2.8-fold) and phosphorylated NF-κB p65 (4.1-fold). These changes correlate with 2–3-fold increases in pro-inflammatory cytokine secretion compared to that in controls (20). Genetic studies employing CRISPR/Cas9-mediated TLR4 deletion attenuate LPS-induced NF-κB activity by 85% (p < 0.001) and suppress downstream inflammatory mediator expression by 70–90% (35, 39). Pharmacological interventions with the selective TLR4 inhibitor TAK-242 (1 μM) block IKKβ phosphorylation, reducing IL-6 and TNF-α production by 62% and 58%, respectively (40). Complementary investigations show that baicalein (10 μM) disrupts MyD88 homodimerization, reducing NF-κB nuclear translocation by 73% (p < 0.01) (41, 42). *In vivo* studies with TLR4-knockout mice demonstrated an approximately 50% reduction in renal macrophage infiltration and an 80% decrease in MCP-1 mRNA levels (p < 0.001) compared with wild-type controls under inflammatory conditions (43, 44). The TLR4/MyD88/NF-κB cascade mediates LPS-triggered inflammatory signaling, as depicted in **Figure 2**.

#### 2.2.3 Clinical implications and therapeutic considerations

The clinical relevance of toll-like receptor 4 (TLR4) signaling is evident in human disease states, particularly in alcohol-related liver pathology. Patients with this condition exhibit a 3–5-fold increase in serum lipopolysaccharide (LPS) concentration (p < 0.001). Hepatic TLR4 mRNA expression is strongly correlated with Child–Pugh scores, suggesting a mechanistic link between bacterial translocation, TLR4 activation, and progressive organ dysfunction (45, 46). Therapeutic interventions targeting this pathway show promise in preclinical studies. For instance, administration of andrographolide (5 mg/kg/day) over 4 weeks attenuates hepatic inflammation, reducing interleukin-6 (IL-6) concentrations by approximately 58% (p < 0.01) through inhibition of MyD88/IKKβ phosphorylation, with accompanying improvements in histological parameters (41, 42). However, TLR4-targeted therapeutics present significant challenges related to homeostatic functions. Prolonged TLR4 suppression increases susceptibility to opportunistic infections 2.3-fold, particularly *Klebsiella pneumoniae*, highlighting the need for selective modulation rather than complete inhibition (47). Recent advances in structural biology have facilitated the development of more sophisticated agents with improved specificity, as exemplified by eritoran (MD-2 binding Kd = 12 nM), which has advanced to Phase II clinical trials for conditions characterized by excessive inflammatory responses (34, 48).

### 2.3 Activation mechanisms of the NLRP3 inflammasome in a multidimensional regulatory network

#### 2.3.1 Molecular activation network

The NLRP3 inflammasome functions as an integrated molecular complex that responds to diverse pathophysiological signals, including exogenous danger signals and endogenous metabolic perturbations. Activation primarily occurs through two convergent pathways initiated by stimuli such as monosodium urate crystals and extracellular ATP (49). The first pathway involves P2X7 receptor engagement, which triggers substantial potassium efflux and a three-fold increase in extracellular potassium concentration. This ionic dysregulation coincides with mitochondrial dysfunction, characterized by elevated production of reactive oxygen species (approximately 250% increase in MitoSOX fluorescence intensity) and permeability transition pore opening, further amplifying cellular oxidative stress (50). Concurrently, a second pathway functions through TLR4/MyD88-dependent signaling, culminating in NF-κB nuclear translocation. This transcriptional activation dramatically enhances NLRP3 expression, with a 34-fold increase in NLRP3 mRNA levels (51, 52). Notably, the gut microbiota potently regulates the sensitivity of the TLR4/MyD88 signaling pathway (observed approximately 2.8-fold differential expression) through the release of metabolites(e.g., hort-chain fatty acids, SCFAs) and structural components (e.g., lipopolysaccharide, LPS). This modulation consequently influences NLRP3 inflammasome transcriptional activation. Clinical studies indicate Bacteroidetes abundance exhibited a negative correlation with TLR4 signaling intensity (r = -0.76, p < 0.01). Conversely, Firmicutes, via the production of butyrate, suppressed NF-κB nuclear translocation efficiency by as up to 42% (53). These mechanisms converge during caspase-1 autocatalytic processing, generating functional p20/p10 subunits that increase three-fold relative to the baseline. Activated caspase-1 catalyzes pro-IL-1β cleavage, resulting in a 40-fold elevation in biologically active IL-1β secretion (54, 55).

#### 2.3.2 Oxidative stress-driven regulatory mechanisms

The interplay between redox homeostasis and inflammasome regulation has been elucidated in experimental models using hexavalent chromium (Cr(VI)). At a concentration of 10 μM, Cr(VI) causes significant mitochondrial dysfunction in intestinal epithelial cells, demonstrated by a 40% decrease in mitochondrial membrane potential (JC-1 fluorescence ratio decreasing from 1.0 to 0.6). This dysfunction is associated with weakened antioxidant defenses, highlighted by a 50% reduction in superoxide dismutase activity and a 200% increase in malondialdehyde levels (p < 0.01) (55, 56). This oxidative milieu markedly enhances inflammasome component expression, as shown by immunoblot analyses indicating a 3.2-fold rise in NLRP3 protein and a 2.8-fold increase in ASC expression, leading to a 400% surge in IL-1β secretion. The NF-κB antagonist BAY11-7082 (5 μM) partially mitigates these effects, decreasing ASC expression by approximately 45% (p < 0.05), underscoring an oxidative stress-inflammatory signaling feedback loop (57, 58).

#### 2.3.3 Emerging paradigms for precision-targeted intervention

Recent studies have introduced new methods to regulate inflammasome activity for potential therapeutic purposes. One such approach involves metabolic modulation using sodium propionate at a concentration of 10 mM, which has shown significant anti-inflammatory effects by reducing renal IL-1β levels by approximately 60%. This SCFA boosts the activity of the nuclear factor erythroid 2-related factor 2 (Nrf2) pathway, leading to an 80% increase in nuclear translocation efficiency and an improvement in the GSH/GSSG ratio from 0.5 to 1.2, indicating restoration of redox homeostasis (58, 59). Another strategy involves disrupting the recognition of selective danger signals using dihydroartemisinin at a dose of 50 mg/kg, which effectively hinders the interactions between high-mobility group box 1 (HMGB1) and the TLR4 receptor with a dissociation constant (Kd) of 3.2 nM. In models of spinal cord injury, this intervention has been shown to decrease tissue IL-1β levels by 45% (p < 0.01) and enhance neurological function, as evidenced by a 38% increase in Basso, Beattie, and Bresnahan locomotor scores compared to control groups (60–62). These findings lay the groundwork for the development of targeted therapeutic approaches against inflammasome-mediated inflammatory conditions, thereby presenting promising avenues for future clinical application.

### 2.4 Pathological networks and targeted reversal of metabolic endotoxemia

#### 2.4.1 Mechanisms of multi-organ damage

**Figure 3:**
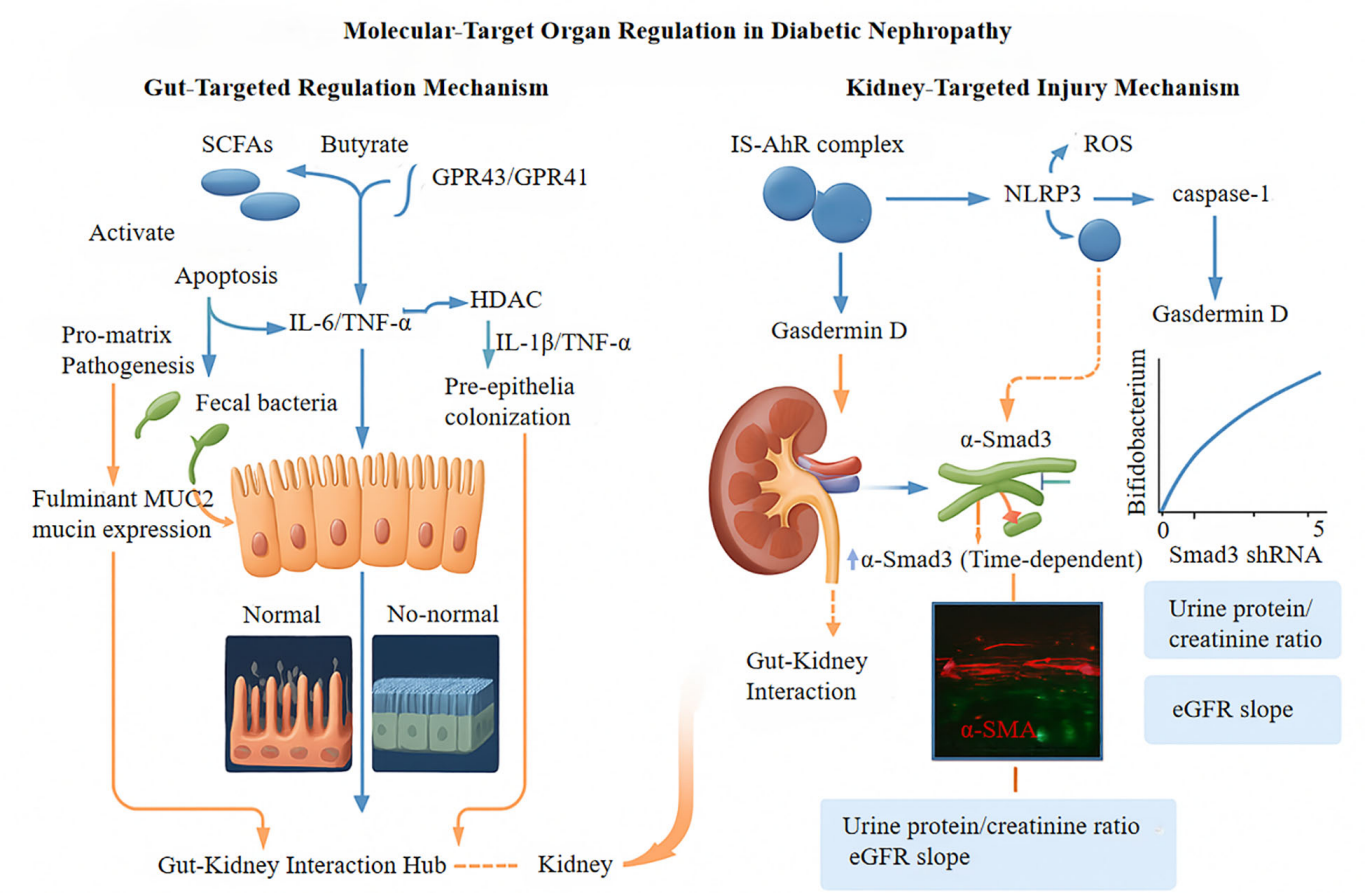
GPR43/GPR41, G Protein-Coupled Receptor 43/41; HDAC, Histone deacetylase; IL-6, Interleukin-6; TNF-α, Tumor Necrosis Factor-alpha; NLRP3, NLR Family Pyrin Domain Containing 3; IS, Indoxyl sulfate; AhR, Aryl hydrocarbon receptor; MUC2, Recombinant Mucin 2; Smad3 shRNA, Gene silencing tool; α-SMA, α-Smooth Muscle Actin; eGFR, Estimated Glomerular Filtration Rate.

Metabolic endotoxemia triggers a complex pathophysiological cascade that results in multi-organ dysfunction through specific cellular and molecular pathways. A key aspect of this process is the metabolic alteration of macrophages via the CD14-TLR4 signaling pathway. Binding of lipopolysaccharide (LPS) to the CD14/TLR4 receptor complex induces significant changes in cellular bioenergetics and inflammatory reactions (63). LPS-induced TLR4 activation boosts glycolytic metabolism by doubling the expression of hexokinase 2 (HK2) (p < 0.01), which is associated with the polarization of pro-inflammatory M1 macrophages. Flow cytometry revealed an increase in the population of inducible nitric oxide synthase-positive (iNOS+) cells from approximately 15% to 65% after endotoxin exposure (p < 0.001) (64, 65). Concurrently, the CD14-TLR4 signaling pathway disrupts mitochondrial function by inhibiting peroxisome proliferator-activated receptor-gamma coactivator-1α (PGC-1α), leading to a 60% decrease in mitochondrial DNA copy number (p < 0.01) and a 50% reduction in cellular ATP production (p < 0.05). This dysfunction results in the accumulation of reactive oxygen species, with mitochondrial superoxide levels increasing to relative light units per milligram of protein, compared to 623 RLU/mg in control samples (p < 0.001) (66, 67). The interplay between heightened glycolysis and impaired mitochondrial oxidative phosphorylation establishes a “mitochondrial-glycolysis metabolic gap” that sustains oxidative stress and pro-inflammatory signaling, contributing to energy metabolism disturbances in various organ systems (68). Molecular targeting and organ regulation in DN are shown in **Figure 3**.

#### 2.4.2 Translational medicine applications

Clinical studies have confirmed the significance of metabolic endotoxemia in human pathophysiology. Research on patients with DN has shown a strong association between serum LPS levels and the urinary protein-to-creatinine ratio. Furthermore, examinations of renal tissue samples from patients compared to healthy individuals have revealed a three-fold increase in the density of TLR4-positive macrophage infiltration compared to healthy individuals (p < 0.001) (69–71). These clinical findings have led to the development of novel, targeted therapeutic strategies.

Luteolin, a flavonoid at a concentration of 20 μM, has demonstrated efficacy in rebalancing macrophage polarization, shifting the M1:M2 ratio from 4:1 to 1:2 (p < 0.01), primarily through activation of the peroxisome proliferator-activated receptor-gamma (PPARγ) pathway (72, 73). Luteolin also significantly improves mitochondrial function, as indicated by the restoration of mitochondrial membrane potential and the recovery of the JC-1 red/green fluorescence ratio from 0.3 to 1.8 (p < 0.001). This protective effect is accompanied by a 72% decrease in NF-κB p65 subunit phosphorylation (p < 0.01), suggesting inhibition of the TLR4/NF-κB inflammatory signaling pathway (74). Furthermore, luteolin enhances the stability of the mitochondrial network by upregulating optic atrophy protein 1 (OPA1) by 1.7-fold (p < 0.05), thereby improving mitochondrial dynamics and resilience against stress-induced fragmentation (75).

These results establish a mechanistic connection between metabolic reprogramming and organ dysfunction in metabolic endotoxemia, highlighting the PPARγ-HK2-PGC-1α signaling network as a crucial regulatory hub with significant therapeutic implications. This molecular pathway is a promising target for the development of comprehensive interventions that address both the inflammatory and metabolic abnormalities associated with endotoxemia (76, 77).

### 2.5 Bidirectional interaction and pathological mechanisms of the gut–kidney axis

The intricate interplay between renal function and intestinal microbiota involves metabolic, immune, and neuroendocrine signaling pathways, which play crucial roles in renal pathophysiology, especially in CKD.

#### 2.5.1 Pathophysiological framework

A decline in renal function leads to changes in the gut microenvironment due to the buildup of nitrogenous waste products. This altered environment exerts selective pressure, reshaping the microbial community by reducing beneficial commensal bacteria such as *Bifidobacterium* and *Lactobacillus* and promoting opportunistic pathogens, especially within the phylum Proteobacteria (78, 79). Consequently, dysbiosis occurs, characterized by an overabundance of gram negative bacteria with increased endotoxin production capacity (80). This disruption is particularly notable in patients with DN. Metagenomic studies have indicated a 30% decrease in alpha diversity indices in patients with DN compared to healthy individuals, with a significant positive association between microbial diversity measures and estimated glomerular filtration rate (eGFR) (7, 81).

#### 2.5.2 Molecular mechanisms of key pathological loops

IS, a protein-bound uremic toxin produced from microbial tryptophan metabolism, hinders enterocyte proliferation by activating the AhR and inhibiting the Wnt/β-catenin signaling pathway. Histological examination indicated a 52% decrease in Ki-67-positive cells in colonic crypts upon exposure to IS (82). Additionally, IS exposure reduces the levels of tight junction proteins (zonula occludens-1 and occludin), leading to a 3.8-fold increase in paracellular permeability (83). This compromised epithelial barrier facilitates the translocation of bacterial endotoxins into systemic circulation, establishing a cycle of “gut dysbiosis, barrier dysfunction, uremic toxin accumulation, and kidney deterioration.” Clinical investigations have revealed a significant association between serum IS levels and urinary albumin-to-creatinine ratios in patients with DN (81, 83).

A complex crosstalk involving Toll-like receptor 4 (TLR4), the NLRP3 inflammasome, and SCFAs underpins the molecular pathogenesis of DN. Ligand-dependent TLR4 activation (e.g., by advanced glycation end-products or LPS), initiates NF-κB-driven pro-inflammatory and fibrotic cascades and NLRP3 inflammasome priming. Oxidative stress-induced NLRP3 inflammasome activation (via TXNIP axis) and mitochondrial dysfunction critically mediates Caspase-1-dependent IL-1β maturation, exacerbating renal damage including mesangial injury, podocyte apoptosis, and tubulointerstitial fibrosis (84).Conversely, gut microbiota-derived SCFAs exert protective effects through multiple mechanisms: direct inhibition of TLR4 signaling (e.g., by attenuating MyD88/NF-κB phosphorylation); modulation of NLRP3 inflammasome activity, partly via GPR43 activation or HDAC inhibition, thereby disrupting essential pathways (e.g., mitochondrial ROS-TXNIP axis) (85). SCFAs also enhance intestinal barrier integrity to reduce systemic endotoxin exposure and thus mitigate TLR4/NLRP3 activation. The observed synergy between SCFAs and NLRP3 inhibitors (e.g., MCC950), potentially involving epigenetic modulation, highlights a novel therapeutic angle. This intricate regulatory network offers a compelling rationale for microbiota-targeted strategies (e.g., probiotic-induced SCFA enhancement) to concurrently dampen TLR4-initiated inflammation and NLRP3-amplified responses, ultimately ameliorating fibrotic remodeling in DN.

#### 2.5.3 Novel interventional strategies and metabolic regulation

Fecal microbiota transplantation (FMT) holds promise for the disruption of pathological circuits. In rodent models of DN, FMT increases the Shannon diversity index of the intestinal microbiota by approximately 40%, leading to significant enrichment of SCFA-producing bacterial genera such as *Roseburia* and *Faecalibacterium*. These alterations are associated with notable enhancements in renal function parameters, including reductions in serum creatinine (25%) and blood urea nitrogen (18%) (86, 87). Gut dysbiosis notably hinders the conversion of primary bile acids to secondary bile acids, resulting in inadequate activation of the farnesoid X receptor (FXR). This disruption affects the FXR-FGF15/19 signaling axis, which governs hepatic glucose and lipid metabolism. Insufficient FXR levels are linked to a 37% decrease in hepatic glycogen content and a 2.1-fold increase in triglyceride accumulation, exacerbating systemic metabolic dysfunction (88, 89). Furthermore, altered microbial metabolites influence renal redox homeostasis by modulating the Keap1/Nrf2/ARE signaling pathway. This leads to reduced expression of crucial antioxidant enzymes, with a 45% decline in renal superoxide dismutase (SOD) activity and a 2.3-fold increase in malondialdehyde concentration (90). This oxidative environment promotes NLRP3 inflammasome activation (a 1.8-fold increase in caspase-1 activity), establishing a self-perpetuating cycle of oxidative stress and inflammation (91).

Future therapeutic strategies should encompass multifaceted approaches targeting the AhR-Wnt signaling axis, refining the FMT donor selection criteria, and exploring combination therapies. Preclinical studies indicate that the concurrent administration of *Clostridium butyricum* and sulforaphane enhances Nrf2 nuclear translocation while decreasing serum IS concentrations (90, 91), offering a more comprehensive therapeutic efficacy for renal disease.

## 3 Targeted interventions for DN via gut microbiota

### 3.1 Mechanisms and applications of probiotic therapy

#### 3.1.1 Immunological homeostasis remodeling

Administration of probiotics has significant immunomodulatory effects and plays a crucial role in renoprotection against DN. A key aspect of these immunological effects is the restoration of T-cell homeostasis, particularly by modulating the regulatory T-cell (Treg) cell/T helper 17 cell (Th17) axis. Specific probiotic strains, such as Lactobacillus LA, reduce Th17-mediated renal inflammatory responses through the intricate regulation of cytokine networks (92). In-depth immunological analyses indicate that probiotic interventions notably decrease the secretion of pro-inflammatory cytokines like interleukin-6 (IL-6), IL-17, and tumor necrosis factor-alpha (TNF-α), while simultaneously increasing anti-inflammatory mediators such as IL-10 and interferon-gamma (IFN-γ). This immunomodulatory impact primarily occurs by inhibiting the Toll-like receptor 4/Nuclear factor-kappa B (TLR4/NF-κB) signaling pathway (93, 94).

Additionally, probiotic supplementation reduces circulating levels of IL-1β, IL-6, and TNF-α while enhancing the IL-10/IL-6 ratio. This collective effect helps correct the Th1/Th2 immune imbalance that is characteristic of DN (95, 96). The activation of peroxisome proliferator-activated receptor-gamma (PPAR-γ) is mechanistically implicated in mediating immunomodulatory effects. PPAR-γ agonists enhance Treg differentiation and function, leading to improved insulin sensitivity by suppressing the pro-inflammatory TNF-α/IL-1β/IL-6 signaling pathway (97). A pivotal study in the *Journal of Clinical Investigation* revealed that probiotics activate the PPAR-γ/CD36 pathway while inhibiting the TLR4/MyD88 pathway, resulting in decreased NF-κB phosphorylation in renal tissues (98, 99). This dual mechanism underlies the immunoregulatory effects of probiotic therapies in DN.

#### 3.1.2 Metabolic reprogramming

SCFAs, particularly butyrate, play a crucial role as metabolic mediators of the beneficial effects of probiotics in DN. These metabolites, originating from microbes, activate G protein-coupled receptors 43 (GPR43) and 41 (GPR41) on intestinal epithelial cells, leading to enhanced PPAR-γ signaling (100). This signaling pathway contributes to enhanced insulin sensitivity and mitochondrial function, which are commonly impaired in DN.

Butyrate effectively reverses the disruption of the intestinal barrier induced by TNF-α/IFN-γ. Functional evaluations have demonstrated the restoration of transepithelial electrical resistance and the upregulation of tight junction proteins such as occludin and zonula occludens-1 (ZO-1) following butyrate treatment (101, 102).

Furthermore, butyrate inhibits HDAC activity, promoting the expression of genes crucial for mitochondrial biogenesis, including peroxisome proliferator-activated receptor-gamma coactivator 1-alpha (PGC-1α) and nuclear respiratory factor 1 (NRF1). This epigenetic modulation enhances energy metabolism in renal tubular epithelial cells (103, 104). Moreover, probiotic interventions lead to a significant increase in circulating SCFA concentrations, particularly those of propionate and butyrate, which enhance insulin signaling by promoting the phosphorylation of insulin receptor substrate-1/phosphoinositide 3-kinase/protein kinase B (IRS-1/PI3K/Akt) through a GPR43-dependent mechanism (96, 105). Metabolic reprogramming is a key mechanism through which probiotic therapy mitigates the progression of DN.

#### 3.1.3 Preservation of the gut–kidney axis

Probiotics maintain intestinal barrier integrity through multiple complementary mechanisms, collectively contributing to the preservation of the gut–kidney axis in DN. Probiotic administration upregulates gene expression and protein abundance of critical tight junction components, including occludin, ZO-1, and claudin-1, directly enhancing intestinal barrier function (92, 103, 106). Furthermore, probiotics repair the intestinal epithelial barrier by modulating the microRNA-29a/Claudin-1 regulatory axis, significantly reducing the translocation of gut-derived uremic toxins, such as IS, into the systemic circulation (104, 107). Probiotic interventions attenuate the release of metabolic endotoxins, including lipopolysaccharide-binding protein (LBP), by downregulating the TLR4/TIR-domain-containing adapter-inducing interferon-β (TRIF) signaling pathway (107, 108). This immunomodulation effectively suppresses the renal expression of IL-17A and monocyte chemoattractant protein-1 (MCP-1), decelerating glomerulosclerosis progression in experimental DN models, as reported by Kidney International (109, 110). Probiotics also counteract the inflammation-induced increases in intestinal epithelial permeability by modulating cytoskeletal regulatory elements, specifically the myosin light chain kinase-myosin light chain (MLCK-MLC) pathway (107, 111). Recent animal studies have demonstrated that probiotic administration inhibits MLCK/phosphorylated-MLC (p-MLC) signaling, thereby reducing intestinal permeability and circulating LBP concentrations. These effects translate to a significant mitigation of renal oxidative stress markers, including reduced malondialdehyde (MDA) levels and improved superoxide dismutase (SOD) activity (103, 112). Furthermore, combined administration of probiotics and dietary fiber enhances the abundance of butyrate-producing bacterial species, particularly *Faecalibacterium prausnitzii*. Through the activation of the butyrate-GPR109A signaling axis, this combination therapy effectively inhibits NOD-like receptor family pyrin domain containing 3 (NLRP3) inflammasome activation and reduces renal IL-18 secretion, providing additional renoprotection in DN (107, 113).

### 3.2 Precision nutritional interventions

Recent studies have revealed the multifaceted mechanisms by which dietary interventions modulate the gut–kidney axis in the management of DN. Resistant starch (RS3), particularly the lotus-derived variant, significantly enriches butyrate-producing bacteria (Faecalibacterium +318%, Roseburia +205%), activating the AMPK/Sirt1/PGC-1α signaling cascade. This activation ameliorates the imbalance in mitochondrial dynamics by modulating the expression of MFN2 and DRP1, ultimately improving renal tubular ATP production (114). Butyrate exerts metabolic benefits through dual epigenetic mechanisms: enhancing renal NAD+/NADH ratios with subsequent class II HDAC suppression and activating GPR43 to promote mitochondrial autophagy (115–117). Complementary fiber components have distinct renoprotective pathways. β-Glucan activates GLP-1 receptors, inducing renal vasodilation with reduced intraglomerular pressure, an effect amplified in FXR rs56163822 T allele carriers. Inulin inhibits the generation of secondary bile acids, particularly taurocholic acid, thereby attenuating TLR4/MyD88/NF-κB inflammatory signaling (114, 116, 118).

Protein metabolic remodeling also shows significant promise. Plant-based protein blends (soy:pea 3:1) reduce uremic toxin buildup by suppressing AhR receptors and enhancing UGT1A9 detoxification (119, 120). Very low-protein diets affect the tryptophan metabolic pathway and restore podocyte autophagy via mTORC1 inhibition. Restricting branched-chain amino acids boosts BCKDH complex activity and mitochondrial efficiency, reducing branched-chain α-keto acid accumulation and oxidative stress (121–124). Therefore, population-specific factors should be considered in clinical applications. Metabolomic models using urinary Neu5Ac and 2-HB effectively predicted low glycemic index intervention responsiveness (125, 126). Serum deoxycholic acid serves as a biomarker for dietary fiber resistance, and co-intervention with colesevelam improves the eGFR in affected patients (127, 128). Asian populations with Prevotella-rich microbiota show better plant protein utilization, but increased urinary oxalate excretion, requiring kidney stone risk monitoring. European groups with Bacteroide-dominant microbiota respond better to whey protein, which is influenced by LCT-13910 C/T polymorphism (120, 129–131).

Future research should focus on 1) integrating spatiotemporal multiomics to understand microbiome-metabolome-epigenome interactions, 2) analyzing gene-environment interactions using GWAS and CRISPR screening, and 3) validating the findings from organoid models to real-world evidence (119, 132). This framework combines mitochondrial dynamics, epigenetic regulation, and microbial metabolic networks into a comprehensive approach to manage DN, advancing precision nutritional medicine beyond current paradigms (115).

### 3.3 Advances in immune regulation through microbiota reconstruction therapy

#### 3.3.1 FMT

Recent research has established critical parameters for optimizing donor selection during FMT. Comprehensive metagenomic analyses indicate that successful “super donors” must exhibit specific microbiological characteristics: a gut microbiota Shannon diversity index exceeding 3.5 (based on consensus gut microbiota diversity criteria where healthy adults demonstrate Shannon indices of 4.0-6.0, and certain studies report values above 3.5 as clinically normal in particular demographics) (32), reflecting robust ecological stability; butyrate synthesis-associated gene clusters comprising more than 0.1% of the total genetic material, supporting metabolic resilience; and a balanced Bacteroidetes-to-Firmicutes phylum ratio between 0.8–1.2, maintaining optimal energy metabolism (133, 134). Notably, FMT from metabolically healthy donors (homeostatic model assessment of insulin resistance, HOMA-IR <2.5) yields significantly greater improvements in recipient insulin sensitivity compared to conventional donors, as evidenced by differential Δ M-values (1.2 vs. 0.4 mg/kg/min). These findings suggest an intricate co-evolutionary relationship between host metabolic phenotypes and microbiota functionality (134, 135).

#### 3.3.2 Molecular pathways of microbiota-organ interaction

In the context of nephroprotection, specific donor microbial signatures, particularly *Akkermansia muciniphila* abundance exceeding 4%, selectively attenuate the excessive activation of the renal TLR4/MyD88/NF-κB inflammatory cascade (P < 0.01). This protective effect is mediated through competitive inhibition of TLR4 receptors by the bacteria-derived Amuc_1100 protein (136, 137). Furthermore, microbiota reconstruction effectively restores indole-3-propionic acid (IPA) metabolic pathways, substantially enhancing podocyte autophagic activity, as demonstrated by a 2.4-fold increase in the LC3-II/LC3-I ratio. This process is fundamentally dependent on the activation of the AhR and subsequent upregulation of the autophagy-associated gene ATG5 (138, 139). Experimental studies in db/db mice show the remarkable efficacy of an 8-week FMT intervention, reducing the glomerulosclerosis index by approximately 57%. Mechanistic investigations have revealed that this therapeutic effect is closely correlated with altered tryptophan metabolism within the gut microbiota, characterized by a metabolic shift from the kynurenine pathway toward serotonin (5-HT) synthesis, resulting in a substantial 63% reduction in the kynurenine/5-HT ratio (139, 140).

#### 3.3.3 Challenges in clinical translation and immune regulatory balance

An initial clinical trial targeting FMT-DN yielded promising results. Single colonoscopic FMT reduced 24-hour urinary protein excretion by 31%, with persistent effects for up to 12 weeks. The increased abundance of the donor-derived bacterium *Dialister invisus* was negatively correlated with recipient serum levels of soluble urokinase-type plasminogen activator receptor (suPAR) (137, 140). However, transient elevation of IL-17A levels in 12% of the participants highlighted the need for a comprehensive assessment of the Th17/Treg balance to monitor immune status (141, 142). Probiotics and FMT entail inherent risks including potential pathogen transmission via donor microbiota transfer and unintended modulation of host immune response, necessitating donor screening and post-treatment immune surveillance (143). Microbiota modulation may raise ethical controversies surrounding the concept of ‘microbial identity.’ The enduring stability of transplanted microbiome and lasting impacts on host-microbe symbiotic relationship remain to be fully elucidated. Within the United States, FMT is regulated as a drug by the Food and Drug Administration (FDA). Although two FMT products, Vowst and Rebyota, have received FDA approval, their indications are currently confined to recurrent Clostridioides difficile infection (rCDI), such as cases of antibiotic-unresponsive colitis and diarrhea, with no provision for use in DN. These limitations partly stem from ethical concerns about long-term effects of microbiota-directed therapies. For example, DN patients often present with comorbid metabolic derangements and immune imbalances. Although FMT holds the potential to indirectly alter disease progression via modifications to the gut-kidney axis, the enduring metabolic and immune sequelae are not yet fully elucidated. To date, no evidence in the literature suggests the FDA has formulated a distinct regulatory pathway for FMT as a treatment for DN. However, significant technical challenges remain in optimizing the viability of therapeutic bacterial communities. The recovery rate of viable bacteria in lyophilized microbiota formulations is currently limited to 45–60%. Recent advances in microencapsulation coating technologies have demonstrated the potential to substantially improve bacterial survival rates by approximately 82% (144, 145). These technological developments provide a robust foundation for the precise application of FMT in the management of immunometabolic disorders.

## 4 Other intervention strategies

In CKD, synbiotics and postbiotics modulate the gut-kidney axis, evidenced by: 2.3-fold increase in butyrate synthesis; improved Treg/Th17 ratio (from 0.52 to 1.27); and activated of PPARγ/Nrf2 pathway. Microencapsulation significantly enhances sustained-release efficacy (e.g., 93% vs. 56% for unencapsulated forms). These interventions regulate gut microbiota, suppress uremic toxins, and reinforce intestinal barrier function, partly through microencapsulated therapies targeting SCFA receptors, which further contributes to improved butyrate synthesis and Treg/Th17 balance (**Table 1**).

**Table 1:** Synergistic immune regulation and precision interventions for renal dysfunction.

Category	Intervention	Mechanism/Component	Key Effects	References
Synbiotics	B. longum BB536 + FOS	Probiotic + Prebiotic synergy	- 2.3-fold ↑ butyrate synthesis (p < 0.01]	(146, 147)
		Dietary fiber interaction	- Inhibits uremic toxin precursors	(141, 142, 148, 149)
		FFAR2/GPR43 activation	- ↑ occludin expression	(150–155)
Postbiotics	Lipoteichoic acid/EPS	TLR2/4-MyD88 pathway modulation	- ↓ CD80/86, ↑ IDO1, ↑ TGF-β/Smad3 in dendritic cells	(151–156)
	Freeze-dried bacteriocins	IL-17A suppression	- 58% ↓ Th17-derived IL-17A (P < 0.001]	(157, 158)
Clinical Outcomes	L. plantarum Lp-115 + GOS	Gut microbiota modulation	- 32 ± 6.8% ↓ serum p-cresol sulfate	(157, 159)
		Butyrate production enhancement	- Fecal butyrate ↑ from 1.9 ± 0.8 to 4.7 ± 1.2 mmol/g	(147, 160)
		Immune balance regulation	- Treg/Th17 ratio ↑ from 0.52 ± 0.15 to 1.27 ± 0.31	(156, 161)
Technology	Microencapsulated butyrate	Sustained-release formulation	- 93% release over 72 h (vs. 56% in probiotics]	(158, 162)
SCFA Therapy	Butyrate (GPR43 agonist]	Epithelial barrier enhancement	- 2.8-fold ↑ occludin/claudin-1 (p < 0.01); 63% ↓ HDAC6-NF-κB	(163, 164)
Nrf2 Activation	KBL577 (propionate derivative]	Mitochondrial ROS suppression	- 2.3-fold ↑ hepatic NQO1 activity	(165, 166)
Toxin Control	AST-120 + ω-3 PUFAs	PPARγ-dependent remodeling	- 41% ↓ IDO1 mRNA	(167–169)
Biomarkers	Serum PAG/IS vs. F/B ratio	Dysbiosis correlation analysis	- Negative correlation with F/B ratio	(170, 171)

Targeted bacteriophage therapy has emerged as a precise approach for modulating the microbiome through the selective lysis of urease-positive pathogens, such as *Klebsiella pneumoniae*. This strategy disrupts aromatic amino acid metabolic pathways while preserving the ecological stability of beneficial SCFA-producing microbiota such as *Roseburia* and *Faecalibacterium*, as evidenced by a 57% reduction in fecal p-cresyl sulfate without significant depletion of beneficial bacteria (172, 173). CRISPR-Cas9-engineered bacteriophages represent a significant technological advancement in microbiome manipulation. By delivering guide RNA targeting tnaA, these phages selectively disable tryptophanase-coding genes in urease-producing *Escherichia coli*, effectively blocking indole biosynthesis and downstream IS production at its metabolic source.

Animal studies confirm a 72% decrease in serum IS levels without any detectable off-target effects (174, 175). In contrast, conventional broad-spectrum antibiotics, which temporarily reduce uremic toxin levels, often cause the irreversible depletion of butyrate-producing bacteria, including *Anaerostipes* and *Eubacterium hallii*. This leads to intestinal barrier impairment and toxin level rebound effects, with post-intervention IS levels reaching 1.8-fold above baseline after 6 weeks (176, 177).

**Figure 4:**
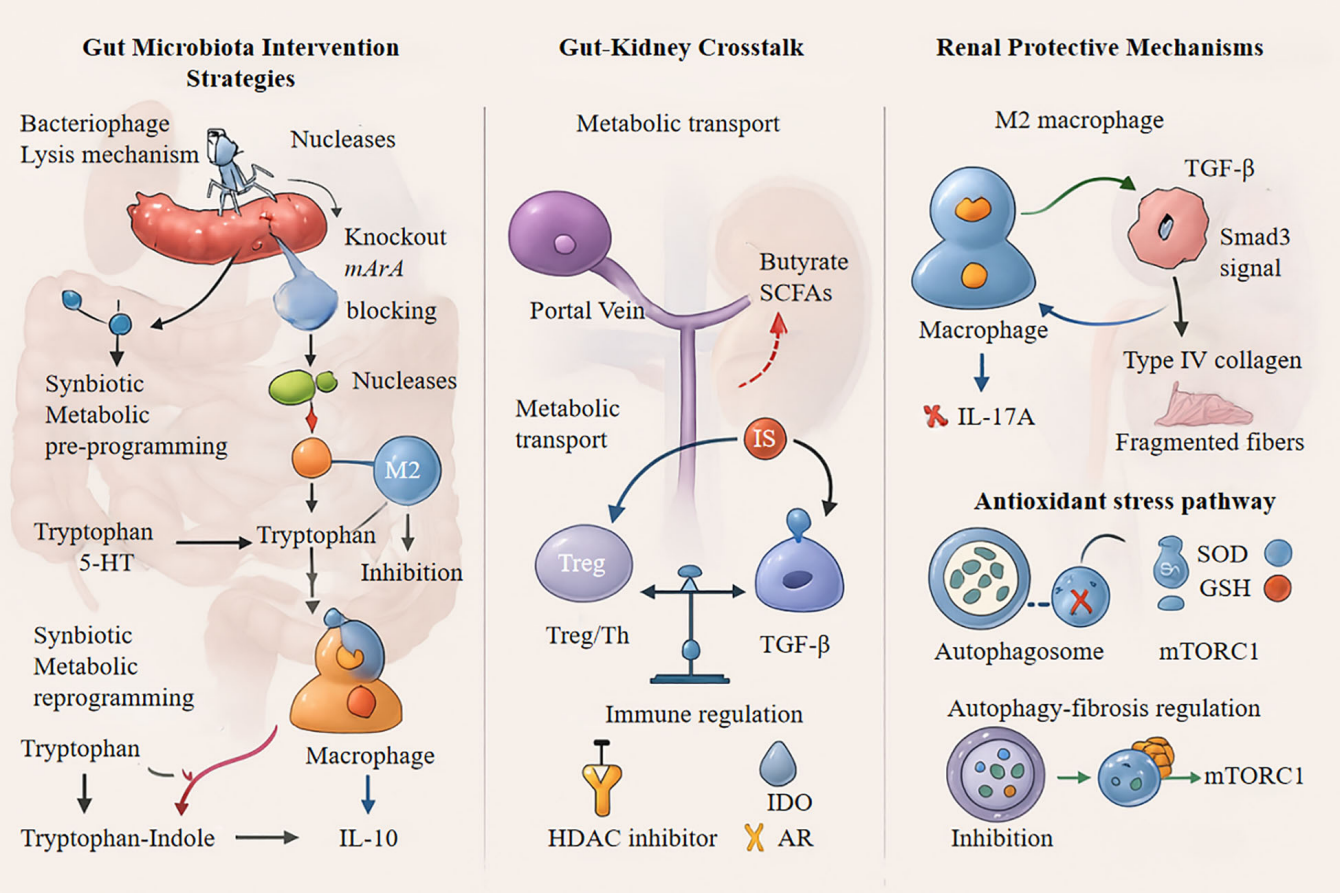
IL-10, Interleukin-10; SCFAs, Short-Chain Fatty Acids; TGF-β, Transforming Growth Factor-beta; HDAC, Histone Deacetylase; IDO, Indoleamine 2,3-Dioxygenase; IL-17A, Interleukin-17A; SOD, Superoxide Dismutase; GSH, Glutathione; MTORC1, Mechanistic Target of Rapamycin Complex 1, Treg, Regulatory T cells.

Sequential bacteriophage-FMT therapy was recently introduced for clinical translation (178). This dual-phase approach, which first eliminates pathogenic bacteria and then reintroduces functional microbiota, successfully reduces the estimated glomerular filtration rate (eGFR) decline by 34% in patients with stage 3 CKD (179, 180). The renoprotective effects significantly correlated with increased gut butyrate concentrations and reduced serum IL-6 levels. These innovations represent a novel paradigm for microbiome-immune axis interventions in the management of CKD progression (**Figure 4**).

## 5 Challenges and future directions

### 5.1 Microbiota therapy challenges: from mechanisms to solutions

Despite promising progress, personalized gut microbiota therapies encounter significant challenges. A major hurdle is inter-individual variability in microbiota composition, which complicates the development of standardized treatment protocols. Approximately 40% of the variability in intervention efficacy is linked to host genotype, dietary habits, and baseline microbiota characteristics (**Figure 3**) (181, 182). Integrating host genome-metabolome-microbiome interaction networks using systems biology approaches significantly enhances the accuracy of efficacy prediction models. A deeper mechanistic understanding is required, especially concerning the molecular pathways through which microbiota-derived metabolites regulate immune cell differentiation.

SCFAs influence these processes via GPR43/GPR41, whereas microbiota-derived tryptophan metabolites modulate the Th17/Treg balance, necessitating further detailed studies (183, 184). Clinical translations face three critical challenges. First, evidence of its long-term safety is limited, with approximately 30% of FMT recipients experiencing immune-related adverse events (181, 185). Second, multiomics techniques require standardization, as combining metagenomics with single-cell metabolomics has uncovered previously undetected functional redundancy at the strain level (186, 187). Regulatory frameworks have not kept pace with technological advancements, which highlights the need for adaptable approval processes based on dynamic microbiota risk assessment (188).

Combined intervention strategies have a significant potential to address these challenges. For instance, a sequential treatment involving probiotics, a Mediterranean diet, and FMT activates the Akkermansia-mediated IL-22/REG3γ pathway, leading to substantial synergistic effects in promoting mucosal healing in models of inflammatory bowel disease (185, 189). These combined approaches offer promising prospects for clinical implementation, and there is a pressing need to develop a comprehensive three-dimensional intervention system that integrates strain tracking, dynamic immune monitoring, and predictive analytics driven by artificial intelligence (190).

### 5.2 Critical barriers to clinical application of FMT

Although FMT shows therapeutic potential for immune-related diseases, its clinical application faces several interconnected challenges. Technical delivery challenges represent the primary barrier. Administration routes significantly impact efficacy, as disease responsiveness varies between lower and upper gastrointestinal delivery approaches. Preparation variables (e.g., fresh vs. frozen samples) affect outcomes though microbial viability variations. Non-standardized manufacturing processes introduce safety concerns, exemplified by FDA warnings on multidrug-resistant organism transmission. The biological interaction between donor and recipient add complexity. Transplant success depends on microbiome compatibility, yet extreme inter-individual variability in immune status, genetics, and baseline microbiota hinders universal donor identification. Clinical evidence showed 30% post-FMT microbiota reversion to baseline suggests host ecosystem resilience may fundamentally limit treatment effectiveness (191). Long-term efficacy presents another significant challenge. Follow-up studies in metabolic disorders revealed that 60% of patients experience diminished metabolic benefits by 12 months post-treatment, with retreatment response rates below 50% of initial therapy (192). This decline may result from competitive exclusion or adaptation of transplanted microbiota in host environments. Standardization deficiencies compound these issues throughout therapeutic pipeline. Inconsistent donor screening protocols, operational variability in preservation media and administration protocols, and the absence of objective biomarker-driven efficacy criteria for complex conditions create significant heterogeneity in clinical practice and outcomes (193). Safety considerations demand careful attention. Although severe adverse events are rare, pathogen transmission risks (e.g., antimicrobial resistance genes, viruses, and hitherto unidentified pathogens) require long-term monitoring, particularly in immunosuppressed patients. Recent research further suggests that FMT may transiently perturb host metabolic or immune homeostasis, as evidenced by reports of extra-intestinal manifestations or autoimmune reactions. To advance FMT from experimental therapy to mainstream treatment requires developing both a comprehensive quality control framework and personalized prediction models based on microbial functional profiles rather than simply compositional data.

## 6 Conclusion

This review examined multiple intervention strategies targeting gut microbiota for the management of DN. Strain-specific probiotic modulation shows substantial potential, particularly when strain selection is guided by metabolic profiles and immune response patterns. Dietary interventions, including strategic fiber supplementation and protein restriction, effectively reshape the microbial composition and function, with evidence supporting their therapeutic efficacy by modulating SCFA and nitrogen metabolism pathways. FMT is the most comprehensive intervention that directly reprograms the gut ecosystem and has shown promising preliminary results in both experimental models and early clinical investigations.

These approaches achieve therapeutic effects through intersecting mechanisms such as restoration of the microbial ecological balance, normalization of microbiota-derived metabolite profiles, and amelioration of gut barrier dysfunction, which contributes to systemic inflammation. The clinical translation of gut microbiota-based therapies faces several fundamental challenges that warrant systematic investigation. First, the relationship between individualized microbiota profiles and treatment responses remains incompletely characterized, with considerable heterogeneity in baseline microbiota composition, confounding standardized approaches. Second, the molecular pathways underlying host-microbiota interactions with specific bacterial strains require further elucidation, particularly regarding the strain-specific effects on immune cell programming and metabolite production. Third, rigorous standardization of methodological approaches is necessary, including donor selection criteria for FMT, therapeutic endpoints, and long-term safety monitoring protocols to detect potential adverse immunological consequences.

Future research should focus on advancing the methodologies across multiple domains. Integrating multiomics technologies such as metagenomics, metabolomics, and single-cell sequencing with advanced organoid models presents unique opportunities to move from correlative associations to causal mechanistic insights. Rigorous multicenter randomized controlled trials with extended follow-up are crucial to validate the renoprotective effects of microbiota-targeted interventions and establish reliable biomarkers to predict treatment responses. These advancements collectively support the shift in diabetic kidney disease management from conventional empirical approaches to evidence-based, microbiome-informed precision medicine, which addresses the complex pathophysiology of the disease through personalized interventions. We urgently call for coordinated international efforts to: 1) Establish open-access biorepositories of multiomics data from diverse patient cohorts; 2) Prioritize organoid-microbiome co-culture systems for mechanistic discovery; 3) Implement adaptive trial designs incorporating real-time biomarker monitoring. These advancements collectively support the shift in diabetic kidney disease management from conventional empirical approaches to evidence-based, microbiome-informed precision medicine.
